# COPI Vesicle Disruption Inhibits Mineralization via mTORC1-Mediated Autophagy

**DOI:** 10.3390/ijms25010339

**Published:** 2023-12-26

**Authors:** Jiaming Nie, Shaoyang Ma, Yuchen Zhang, Shuchen Yu, Jiajia Yang, Ang Li, Dandan Pei

**Affiliations:** Key Laboratory of Shaanxi Province for Craniofacial Precision Medicine Research, College of Stomatology, Xi’an Jiaotong University, Xi’an 710004, China

**Keywords:** COPI vesicle, autophagy, mineralization

## Abstract

Bone mineralization is a sophisticated regulated process composed of crystalline calcium phosphate and collagen fibril. Autophagy, an evolutionarily conserved degradation system, whereby double-membrane vesicles deliver intracellular macromolecules and organelles to lysosomes for degradation, has recently been shown to play an essential role in mineralization. However, the formation of autophagosomes in mineralization remains to be determined. Here, we show that Coat Protein Complex I (COPI), responsible for Golgi-to-ER transport, plays a pivotal role in autophagosome formation in mineralization. COPI vesicles were increased after osteoinduction, and COPI vesicle disruption impaired osteogenesis. Mechanistically, COPI regulates autophagy activity via the mTOR complex 1 (mTORC1) pathway, a key regulator of autophagy. Inhibition of mTOR1 rescues the impaired osteogenesis by activating autophagy. Collectively, our study highlights the functional importance of COPI in mineralization and identifies COPI as a potential therapeutic target for treating bone-related diseases.

## 1. Introduction

Bone mineralization is the process through which calcium phosphate crystals are deposited onto collagen fibrils, which is indispensable to the mechanical support of body mass and protection of the internal soft organs [1]. Bone homeostasis is a process comprising dynamic balances of osteoclasts and osteoblasts, which promote the resorption and formation of bones, respectively [2]. An imbalance in bone homeostasis results in enhancement or loss of mineralization, such as osteopetrosis and osteoporosis. According to recent statistics from the International Osteoporosis Foundation, more than 200 million people worldwide suffer from osteoporosis, a disease characterized by low bone density [3]. Bone mineralization is a universal and sophisticated process regulated by physiological or pathological processes such as proliferation [4], oxidative stress [5], and autophagy [6].

Autophagy, a process of clearing damaged proteins and organelles inside the cell [7], has been proven to regulate bone mineralization [6,8,9]. Additionally, autophagy in osteoblasts is crucial for maintaining bone mass by balancing bone homeostasis [2,10]. Appropriate autophagy level in osteoblasts is a prerequisite for maintaining osteogenic differentiation [11], mineralization [6], and survival [2,12]. Autophagy is actively involved in osteoblast-mediated new bone formation, such as TGF-β and BMP signaling [13,14]. The most direct evidence of the role of autophagy in mineralization is the identification of mineralization precursors in autophagy vacuoles [6]. Our previous research found that autophagy level is increased after osteogenic induction leads to an increased level of mineralization-related proteins RUNX2 and ALP [15,16], and the inhibition of autophagy activity results in the suppression of osteogenesis [17]. However, the reasons for this increase in autophagy levels during mineralization remain obscure.

Autophagy begins with autophagosome formation [18], and the membrane source of autophagosomes has the potential to determine autophagy level. The Golgi apparatus, responsible for the proper processing of proteins, is an important membrane source for autophagosome formation [19]. The Golgi apparatus uses a protein-coated vesicle, namely a Coat Protein Complex I (COPI) vesicle, to manage cargo traffic from the Golgi to the ER or between different compartments of the Golgi [20]. The function of the COPI vesicle is dependent on coatomer subunits, which are composed of seven subunits, found in two subcomplexes, F-COPI (β, γ, δ and ζ) and B-COPI (β’, α and ε) [21]. It has been reported that depletion of COPI subunits (α, β, β’, γ or δ) resulted in the accumulation of autophagosomes and inhibition of autophagy in mammalian and plant cells, respectively [22,23]. Another recent work showed that COPI-ζ1 depletion promoted autophagy in glioblastoma cells [24]. The above studies indicate that COPI vesicles regulate the fusion of autophagosomes and early endosomes. However, the function of COPI vesicles in autophagosome formation and autophagy-regulated mineralization remains largely enigmatic.

Rapamycin was initially found to be an antifungal antibiotic produced by *Streptomyces hygroscopicus* [25]. mTOR (mechanistic target of rapamycin kinase) is one of the most critical pathways that negatively regulate autophagy in mammalian cells [26], and forms two distinct functional complexes, i.e., mTOR complex 1 (mTORC1) and mTORC2. mTOR also plays a crucial role in regulating autophagy in many ways [27]. mTORC1 inhibits ULK1-dependent autophagy by phosphorylating ULK1 at serine 757 [28]. mTORC1 also suppresses autophagy by phosphorylating and inhibiting nuclear translocation of the transcription factor EB, which promotes the transcription of lysosomal biogenesis and the autophagic process [29]. Recently, the regulatory function of mTORC1 in mineralization has been receiving much attention. Deletion of Raptor, a unique and essential component of mTORC1, resulted in a decrease in matrix synthesis and mineralization [30]. The AMPK/mTOR signaling pathway promotes osteogenic differentiation by activating autophagy [31]. 

Here, we provided an unambiguous demonstration of the regulatory function of COPI vesicles in autophagy. Disruption of COPI vesicles decreased autophagy and inhibited mineralization. Mechanistically, the COPI vesicle functions upstream of mTORC1 and activates autophagy by regulating the phosphorylation of S6K1. To the best of our knowledge, this is the first study to demonstrate the function of COPI vesicles in mineralization, which deepens understanding of the regulatory mechanisms during mineralization and offers a possible therapeutic target for osteoporosis.

## 2. Results

### 2.1. COPI Vesicles Are Disrupted in Osteoporosis

**Figure 1:**
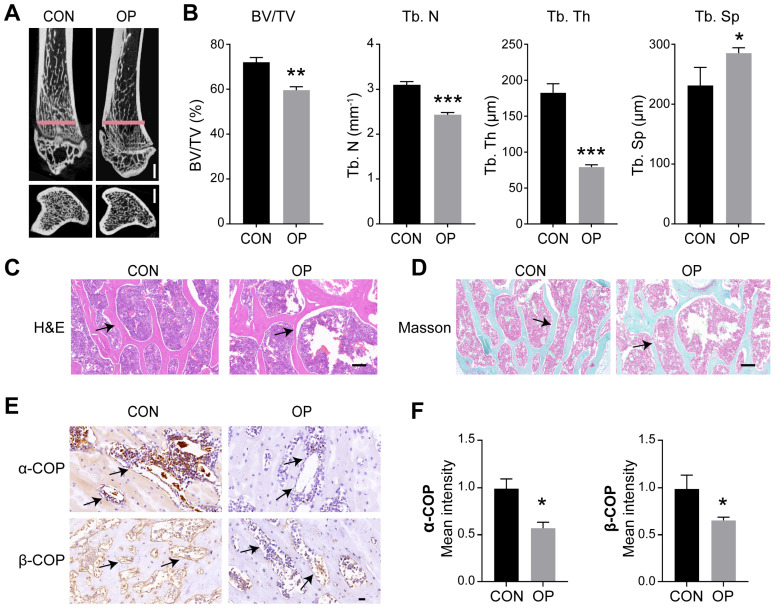
COPI vesicles are decreased in osteoporosis. (**A**,**B**) Representative micro-CT images (**A**) and quantitative analysis of bone mass and bone microarchitecture parameters (**B**) in tibia from CON (control) and OP (osteoporosis) rats. Scale bar: 1 mm. *n* = 3 per group. BV/TV, bone volume/tissue volume; Tb. Th, trabecular thickness; Tb. N, trabecular number; Tb. Sp, trabecular separation. Scale bar: 2 mm. (**C**,**D**) H&E staining (**C**) and Masson staining (**D**) of tibia from the CON and OP rats. Scale bar: 500 μm. Arrows: bone trabecula. (**E**,**F**) Representative immunohistochemical images of proximal tibia sections’ staining with COPI proteins (α-COP and β-COP) and quantitative analysis. Scale bar: 20 μm. *n* = 3 per group. Arrows: osteoblasts. Data are means ± SD, and *p* values were quantified using *t* test. * *p* < 0.05; ** *p* < 0.01, *** *p* < 0.001.

Osteoporosis (OP) is a common bone disease in which decreased bone mass encounters reduced autophagic activity [32,33,34], which is alleviated by autophagy activation [18,35] and exacerbated by autophagy suppression [36]. We constructed an osteoporosis rat model by administering retinoic acid and scanned the tibia by microcomputed tomography (micro-CT) (Figure 1A). The analysis showed less bone mass and impaired microarchitecture in the OP group: BV/TV (bone volume per tissue volume), Tb. Th (trabecular thickness) and Tb. N (trabecular number) were, respectively, 12%, 18% and 41% lower and Tb. Sp (trabecular separation) was 48% higher (Figure 1B). Additionally, histological analyses, including H&E and Goldner staining, showed decreased levels of mineralization in the trabecular bone of the tibia, illustrated as thickness and number (Figure 1C,D). These results show that the osteoporosis model was established successfully.

To detect the changes in COPI vesicles in osteoporosis, we performed immunohistochemistry of the tibia of osteoporosis rats. The results revealed that the levels of COPI biomarkers (α-COP and β-COP) significantly decreased (Figure 1E,F). The above results showed that COPI vesicles decreased in osteoporosis, which suggested a close relationship between the COPI vesicles and mineralization.

### 2.2. BFA-Induced COPI Vesicle Disruption Inhibits Mineralization

**Figure 2:**
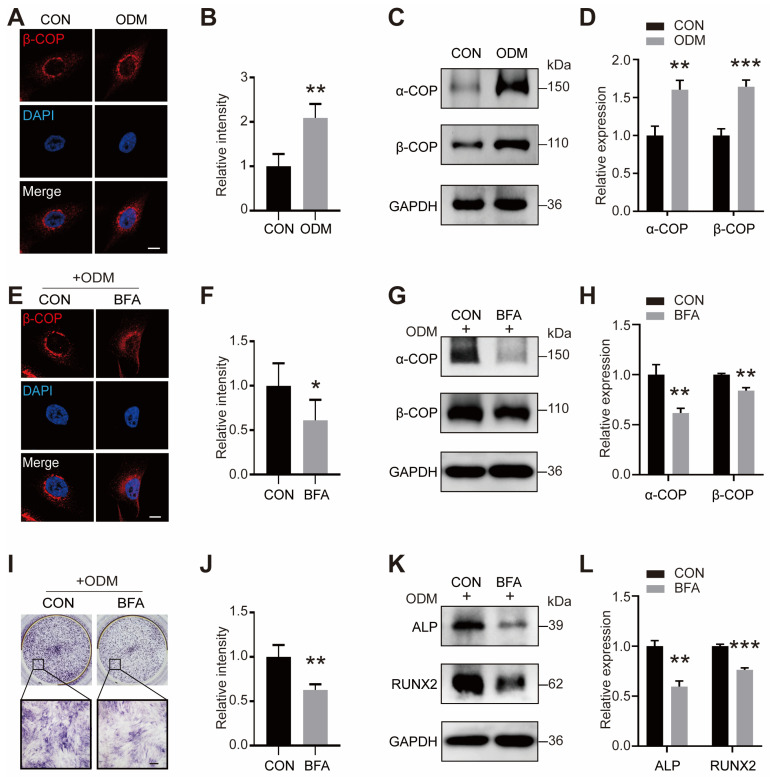
BFA-induced COPI vesicle disruption inhibits osteogenic effect. (**A**,**B**) Representative immunofluorescence images staining for β-COP (red) and the quantitative analysis of β-COP expression in control (CON) or osteogenic differentiation medium (ODM) condition for 3 d. The nucleus was highlighted by DAPI. Scale bar: 5 μm. *n* = 5 per group. (**C**,**D**) Western blot of COPI proteins (α-COP and β-COP) and quantitative analysis. *n* = 3 per group. (**E**,**F**) Representative immunofluorescence images staining for β-COP (red) and the quantitative analysis of β-COP expression with or without Brefeldin A (BFA) in ODM condition for 3 d. The nucleus was highlighted by DAPI. Scale bar: 5 μm. *n* = 5 per group. (**G**,**H**) Western blot of COPI proteins (α-COP and β-COP) and quantitative analysis. *n* = 3 per group. (**I**,**J**) ALP staining (**I**) and the quantitative analysis (**J**). Scale bar: 150 μm. *n* = 3 per group. (**K**,**L**) Western blot of mineralization-related proteins and quantitative analysis. *n* = 3 per group. Data are means ± SD, and *p* values were quantified using *t* test. * *p* < 0.05; ** *p* < 0.01, *** *p* < 0.001.

To gain insight into the effect of COPI vesicles on mineralization, we first tested the level of COPI vesicles during osteogenic induction. The immunofluorescence images’ staining and Western blot showed that the expression levels of α-COP and β-COP were increased when cells were exposed to the osteogenic differentiation medium (ODM) condition (Figure 2A–D). We found elevated levels of LC3 II and BECLIN1, indicating that autophagy activity is enhanced after osteogenesis (Figure S1A,B). Then, we treated the cells with Brefeldin A (BFA, Figure S2A) to block the assembly of COPI coats. Consistent with prior studies [37], BFA treatment switched the distribution of COPI vesicles from perinuclear distribution to dispersed distribution throughout the cytoplasm (Figure 2E), and decreased the level of COPI vesicles (Figure 2E–H). Runx2 and ALP are important indicators of mineralization and have a critical function in the formation of hard tissue [15,16]. Moreover, BFA treatment reduced the levels of ALP staining and osteogenesis-related proteins (ALP and RUNX2) (Figure 2I–L). The above observations showed that BFA-induced COPI vesicle disruption impaired the mineralization of MG63 cells. 

### 2.3. COPI Disruption Results in Impaired Autophagy Flux

**Figure 3:**
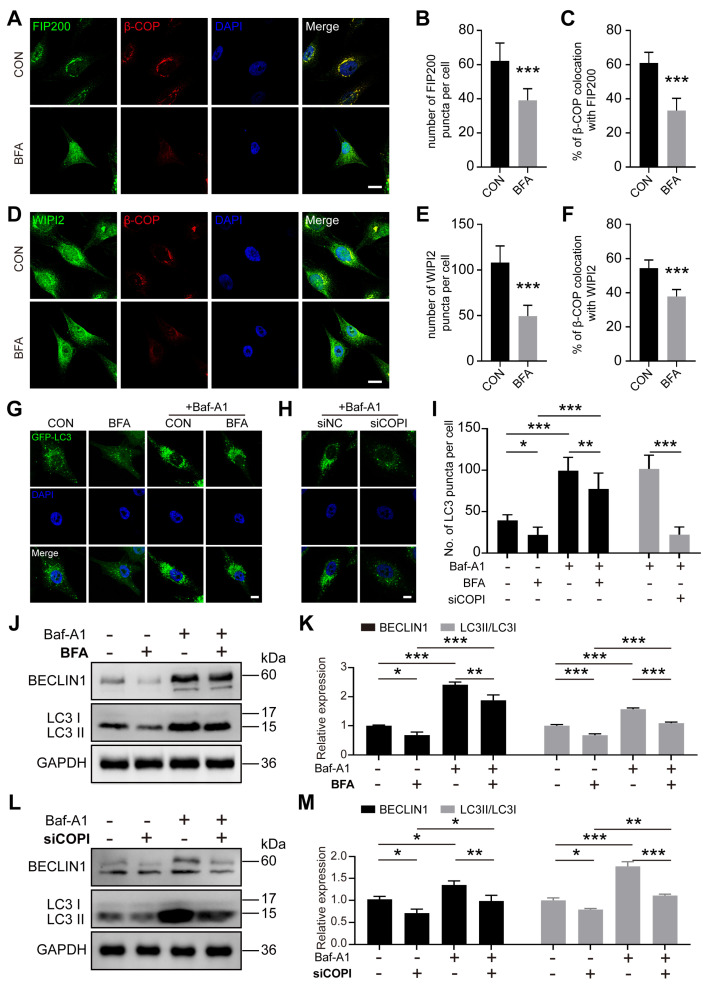
COPI dysfunction results in impaired autophagy flux. (**A**–**C**) Representative immunofluorescence images staining for FIP200 (green) and β-COP (red), the number of FIP200 puncta (**B**) and the percentage of FIP200 puncta colocation with β-COP puncta (**C**). Scale bar: 10 μm. *n* = 10 per group. (**D**–**F**) Representative immunofluorescence images’ staining for WIPI2 (green) and β-COP (red), the number of WIPI2 puncta (**E**) and the percentage of WIPI2 puncta colocation with β-COP puncta (**F**). Scale bar: 10 μm. *n* = 10 per group. (**G**–**I**) Representative immunofluorescence images of GFP-LC3 plasmid assay of BFA-treated cells and siCOPI cells and the quantitative analysis of LC3-positive puncta (**I**) with or without Bafilomycin A1 (Baf-A1). Scale bar: 5 μm. *n* = 10 per group. (**J**,**K**) Western blot and quantitative analysis of autophagic proteins (BECLIN1 and LC3) in BFA-treated cell. *n* = 3 per group. (**L**,**M**) Western blot and quantitative analysis of autophagic proteins (BECLIN1 and LC3) in siCOPI cells. *n* = 3 per group. Data are means ± SD, and *p* values were quantified using *t* test and one-way ANOVA with Tukey’s post hoc test. * *p* < 0.05; ** *p* < 0.01, *** *p* < 0.001.

To explore whether COPI participates in the autophagy process. we examined reporters for autophagic structures at different stages in MG63 cells. The ULK1/FIP200/ATG13 complex labels the autophagosome formation sites on the ER [38,39]. The PtdIns(3)P-binding protein WIPI2 can label isolating membranes but not closed autophagosomes [40]. The number of puncta formed by FIP200 or WIPI2 was dramatically decreased under BFA treatment (Figure 3A,B,D,E). Meanwhile, the colocalizations between FIP200 and β-COP or WIPI2 and β-COP became looser than the control cells (Figure 3A,C,D,F). In addition, we labeled LC3, a key biomarker of autophagosomes [41], using GFP-LC3 plasmids, and then we detected the immunofluorescences. Bafilomycin A1 (Baf-A1) has been confirmed to be a specific inhibitor of autophagy by blocking fusion between autophagosome and lysosome [7]. Baf-A1 (50 nM) was used in the following experiments (Figure S2C). Our results showed that the expression of β-COP mRNA decreased in siCOPI cells (Figure S3A). The levels of GFP-LC3 puncta unsurprisingly increased upon Baf-A1 treatment in the absence or presence of BFA, suggesting Baf-A1 functions (Figure 3G,I). There is a baseline level present in MG63 cells without any treatment. BFA treatment resulted in decreased levels of LC3 puncta without Baf-A1. Upon Baf-A1 treatment, BFA-treated cells also inhibited the levels of LC3 puncta (Figure 3G,I). Furthermore, the deficiency of COPI by siRNA decreased the levels of LC3 puncta with Baf-A1 (Figure 3H,I). Next, we further explored the level of autophagy-related proteins (BECLIN1 and LC3II) to verify the effect of COPI inhibition on autophagy. Consistent with the GFP-LC3 result, levels of LC3-II and BECLIN1 decreased when cells were exposed to BFA with or without Baf-A1 treatment (Figure 3J,K). The basal levels of BECLIN1 and LC3II were further reduced without Baf-A1 treatment in BFA-treated cells, and BFA led to reduced expression of LC3-II and BECLIN1, although Baf-A1 generated the anomaly accumulation of those proteins. The deficiency of COPI by siRNA also decreased the levels of LC3-II and BECLIN1 with or without Baf-A1 treatment (Figure 3L,M). In addition, the levels of autophagic biomarkers (BECLIN1 and LC3) significantly decreased in osteoporosis (Figure S4A,B). Those discoveries suggest that autophagosome formation is restrained by COPI interference.

### 2.4. COPI Affects Autophagy via the mTORC1 Pathway

**Figure 4:**
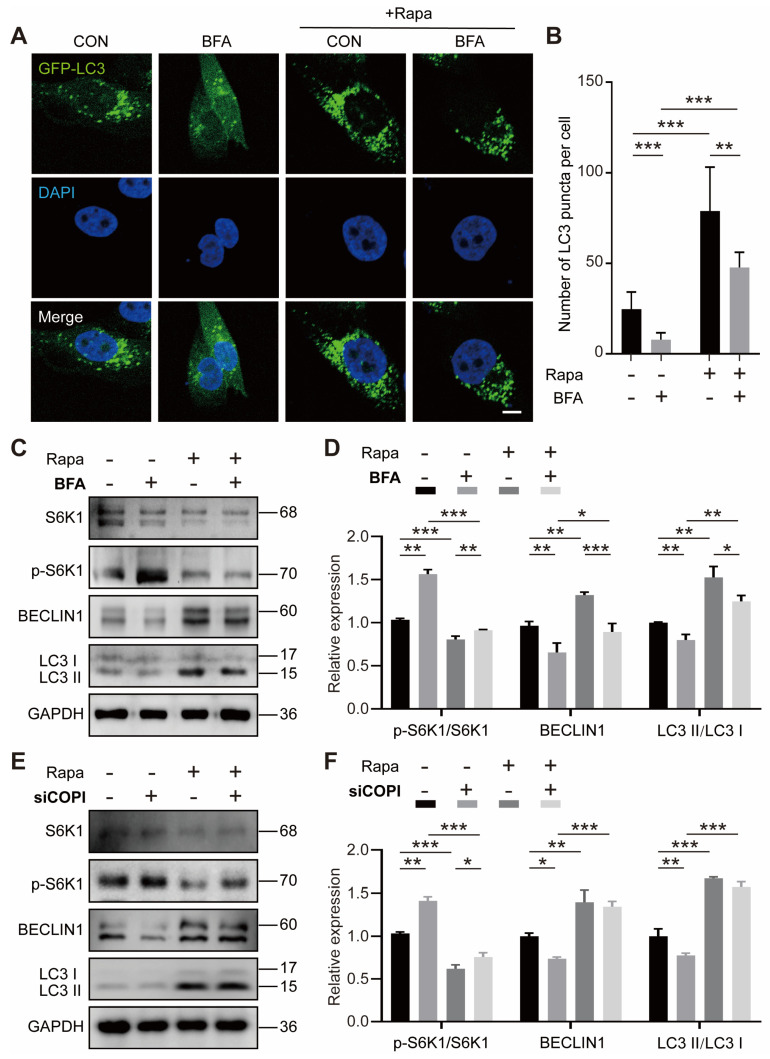
COPI affects autophagy via the mTORC1 pathway. (**A**,**B**) Representative immunofluorescence images of GFP-LC3 plasmid assay (**A**) and the quantitative analysis of LC3-positive puncta (**B**) with or without rapamycin (Rapa). Scale bar: 5 μm. *n* = 10 per group. (**C**,**D**) Western blot and quantitative analysis of autophagic proteins in BFA-treated cells. *n* = 3 per group. (**E**,**F**) Western blot and quantitative analysis of autophagic proteins in siCOPI cells. *n* = 3 per group. Data are means ± SD, and *p* values were quantified using one-way ANOVA with Tukey’s post hoc test. * *p* < 0.05; ** *p* < 0.01, *** *p* < 0.001.

As mTORC1 is a key regulatory factor of autophagy [42], we speculated that the mTORC1 pathway determined the effect of COPI vesicles on autophagy. To verify this speculation, we treated the osteogenic-induced MG63 cells with rapamycin, an antifungal antibiotic from *Streptomyces hygroscopicus*, and a specific inhibitor for mTORC1. Rapamycin (Figure S2B) was used in the following experiments. The results of the GFP-LC3 immunofluorescence images showed that BFA treatment reduced the number of LC3 puncta (~68% lower in the absence of rapamycin and ~40% lower in the presence of rapamycin (Figure 4A,B)), suggesting that inhibition of mTORC1 ameliorates the suppression of COPI disruption on autophagy activity. Then, we detected the expression of p-S6K1 (a biomarker of mTOR activation) in MG63 cells under osteoinduction and found that p-S6K1 expression increased by 51% upon BFA treatment in the absence of rapamycin, while rapamycin treatment restored this increase by 12% (Figure 4C,D). Additionally, the BFA-induced COPI disruption decreased BECLIN1 and LC3II levels by 32% and 20%, respectively, and rapamycin partially restored the downtrend (Figure 4C,D). Furthermore, our results showed that COPI disruption by siCOPI increased the levels of p-S6K1 by 38% in the absence of rapamycin, while rapamycin restored the increase (Figure 4E,F). In addition, COPI disruption by siCOPI also decreased BECLIN1 and LC3II levels by 26% and 22%, respectively, and rapamycin completely restored the downtrend of LC3II (Figure 4E,F). The above results indicate that the mTORC1 pathway is involved in COPI vesicle–autophagy interplay. 

### 2.5. Inhibition of mTORC1 Rescues the Suppression of COPI Vesicle Disruption on Mineralization

**Figure 5:**
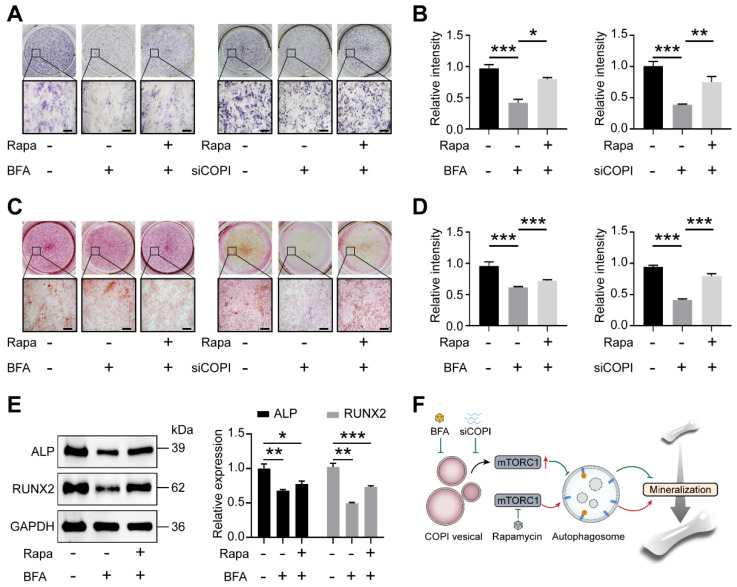
Inhibition of mTORC1 partially rescues the suppression of COPI vesicle disruption of osteogenic effects. (**A**,**B**) ALP staining in BFA-treated cells and siCOPI cells (**A**) and the quantitative analysis (**B**). Scale bar: 75 μm. *n* = 3 per group. (**C**,**D**) Alizarin red-S staining in BFA-treated cells and siCOPI cells (**C**) and the absorbance values of alizarin red-S staining were measured at OD_560nm_ (**D**). Scale bar: 75 μm. *n* = 3 per group. (**E**) Western blot and quantitative analysis of osteogenic proteins ALP and RUNX2 in BFA-treated cells. *n* = 3 per group. (**F**) Schematic diagram of the role of COPI on autophagy in mineralization and rapamycin-rescued system. Data are means ± SD, and *p* values were quantified using one-way ANOVA with Tukey’s post hoc test. * *p* < 0.05; ** *p* < 0.01, *** *p* < 0.001.

We next sought to investigate whether inhibition of mTORC1 by the COPI vesicle could affect the mineralization of MG63 cells. Western blot results showed that the expressions of osteogenesis-related proteins (ALP and RUNX2) were significantly decreased under the treatment of BFA (Figure 5A,B). However, after the addition of rapamycin, the expressions of ALP and RUNX2 partially recovered (Figure 5A,B). Additionally, the activity of ALP, an early osteogenic marker, was significantly decreased in MG63 cells treated with BFA or by siCOPI while nearly returning to baseline after rapamycin treatment (Figure 5C,D). Similarly, the inhibition of alizarin red S staining by BFA or by siCOPI was partially restored by rapamycin (Figure 5E,F). The above observations suggest that COPI regulates osteogenic capacity in an autophagy-dependent way.

### 2.6. Knockdown of COPI Results in Bone Loss and Decreased Autophagic Activity in Osteoporosis Rats

**Figure 6:**
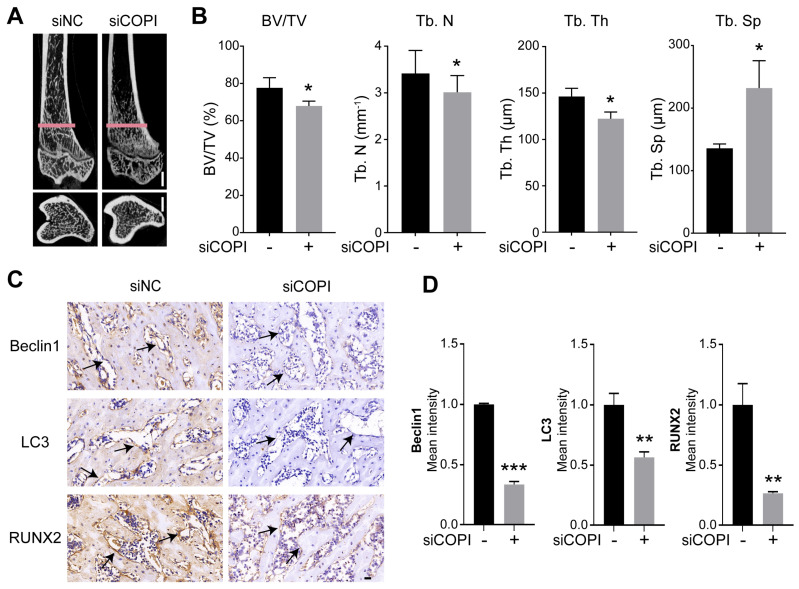
Knockdown of COPI results in bone loss and decreased autophagic activity. (**A**,**B**) Representative micro-CT images (**A**) and quantitative analysis of bone mass and bone microarchitecture parameters (**B**) in tibia from siNC and siCOPI rats. Scale bar: 1 mm. *n* = 3 per group. BV/TV, bone volume/tissue volume; Tb. Th, trabecular thickness; Tb. N, trabecular number; Tb. Sp, trabecular separation. Scale bar: 2 mm. (**C**,**D**) Representative immunohistochemical images of proximal tibia sections’ staining with autophagic proteins (Beclin1 and LC3) and mineralization-related protein RUNX2 and quantitative analysis. Scale bar: 20 μm. *n* = 3 per group. Arrows: osteoblasts. Data are means ± SD, and *p* values were quantified using *t* test. * *p* < 0.05; ** *p* < 0.01, *** *p* < 0.001.

To detect the effect of COPI dysfunction on mineralization and autophagic activity in osteoporosis rats, we performed siCOPI transfection. The analysis of micro-CT showed that knockdown of COPI resulted in bone loss, demonstrated as decreased levels of BV/TV, Tb. N and Tb. Th and increased levels of Tb. Sp (Figure 6A,B). We found that the levels of autophagic biomarkers (Beclin1 and LC3) and mineralization-related protein RUNX2 significantly decreased in siCOPI rats (Figure 6C,D). The above results showed that knockdown of COPI led to bone loss and decreased levels of autophagic activity.

## 3. Discussion

In this study, we examined the critical role of the COPI vesicle, required at multiple stages of ER-to-Golgi transport [43], in regulating mineralization via mTORC1-mediated autophagy. COPI vesicle disruption decreased mineralization level via impaired autophagy activity. These findings provide a new perspective for autophagy activity in mineralization.

Bone matrix mineralization is a tightly regulated process and requires the coordination of multiple cellular organelles, including Golgi. It has been reported that the Golgi protein SLC10A7, a genetic factor for Golgi homeostasis and glycoprotein trafficking, regulated bone mineralization by influencing glycosylation and transport of proteoglycans and glycoproteins to the extracellular matrix [44]. COPI vesicles are responsible for the retrograde transport of proteins from the Golgi to the ER and for intra-Golgi transportation [45]. In the present research, we found that suppression of COPI vesicle formation by BFA treatment leads to the inhibition of mineralization (Figure 2), which revealed the function of COPI vesicles in mineralization for the first time. Our findings provide novel evidence for the regulation of mineralization by Golgi.

Coated vesicles (COPI, COPII, and clathrin) are responsible for trafficking proteins throughout the endomembrane system [45]. COPII vesicles mediate the anterograde transport from ER to Golgi, while clathrin-coated vesicles transfer proteins out of the trans-Golgi network or transfer materials endocytosed from the cell surface [46]. In addition, studies have reported that coated vesicles are involved in autophagy. In the present work, we found that BFA treatment led to COPI disruption (Figure 2), and that the expression of FIP200 and WIPI2 (markers of the early stages of autophagy) and LC3 (a key marker of autophagosomes) decreased in BFA-treated MG63 cells (Figure 3). The lower percentage of colocalization of COPI element with FIP200 and WIPI2 illuminated that the autophagy flux was prevented in the early stage and that autophagosome formation was restrained in the following order. Additionally, we found that the inhibition of mTORC1 ameliorated the suppression of COPI disruption on autophagy activity (Figure 4). The above results suggest that COPI disruption results in the inhibition of autophagy, which is involved in the mTORC1 pathway. In addition, the COPI vesicle possesses multiple functions, such as Golgi–ER trafficking [43], mitochondria–ER contact site formation [47], reactive oxygen production [48], and cell differentiation [49]. It has been reported that mitochondria–endoplasmic reticulum contact sites are involved in the formation of autophagosomes [50], which need further exploration.

COPII vesicles regulated autophagosome formation in *Saccharomyces cerevisiae* under starvation conditions by interacting with ATG9 vesicles [51]. Another study showed that COPII vesicles contributed to autophagosomal membranes, which regulated the formation of autophagosomes [52]. Recently, the regulation of COPI vesicles in autophagy has been gaining traction. However, studies on COPI vesicles in autophagy have focused on cargo transport and the fusion of COPI vesicles with early endosomes [22]. Here, we found that the colocalization of COPI vesicles and markers of the early step of autophagosome formation increased under mineralization, which was abolished by BFA treatment (Figure 3). Our data deepen understanding of the mechanism of autophagosome formation during mineralization, in which COPI vesicles are involved.

Besides mediating autophagosome formation, COPI vesicles in our study may also regulate autophagy levels by transporting proteins of the mTOR pathway, which is crucial in regulating autophagy [53]. In the present research, we found that mTORC1 activity was increased by BFA treatment (Figure 4). There have been a number of reports identifying that mTORC1 and other components associated with the activation of mTORC1 are closely linked to Golgi, and some Golgi membrane tethers/scaffolds that directly or indirectly regulate mTORC1 activation have been identified [54,55,56]. Thus, the inhibition of mTORC1 ameliorated the suppression of COPI vesicle disruption of osteogenic effect (Figure 5). The above studies, combined with our results, indicate a possible mechanism where the transport of those Golgi-located mTORC1 signaling proteins is regulated by COPI vesicles, resulting in changes in autophagy level and mineralization. 

BFA is a small hydrophobic compound produced by *Eupenicillium brefeldianum*, and BFA exerts a disruptive effect on the assembly of the COPI by inactivating Arf1-GTP [37,57]. BFA, owning to its destructive effect on COPI vesicles, has been widely applied in monkey fetal kidney MA104 cells [58], Madin–Darby canine kidney cells [59], mouse embryonic fibroblast NIH3T3 cells [60], Nicotiana tabacum BY-2 cells [61], mouse macrophage J774-E cells and human hepatocellular carcinoma HepG2 cells [62]. In this study, our results found that autophagy activity was decreased in BFA-treated osteoblasts (Figure 3). Furthermore, we found that the expression of autophagic markers at the early stage reduced, suggesting that BFA-induced autophagy was hindered in the early phase. In addition, BFA exerts a negative effect on autophagosomal maturation by preventing the entry of the Golgi marker GM130 and chymotrypsin into the LC3-II-rich compartments in acute pancreatitis [63]. On the contrary, BFA amplifies autophagic activity to realize the goal of antitumor activity by promoting tumor cell death [64,65]. Thus, our findings, together with these previous studies, indicate that BFA effects on autophagic activity remain complicated and diverse, which needs to be further explored in the future. 

There are some aspects to consider that could be improved in the present study. MG63 cell is a typical osteoblast [66,67,68], and it has been commonly used for cellular mechanism studies about osteogenesis [69,70,71]. However, applying multiple cell lines and primary cells helps solidify the relationship between COPI vesicles and mineralization. Our observations led to some interesting preliminary conclusions about the role of COPI vesicles in the formation of early autophagosomes for the first time. Nevertheless, how protein interactions [51], lipid transfers [72,73], and other novel strategies related to COPI vesicles are involved in autophagosome formation needs to be further explored. Additionally, we revealed that COPI vesicles regulated the mTORC1 pathway, but the detailed molecular mechanism remains to be clarified in future studies. It has been reported that COPI/II vesicles transport mineralized secreted proteins such as collagen [74] and ALP [16] in osteoblasts. The depletion of COPI complex subunits leads to the accumulation of reactive oxygen species (ROS) and apoptosis of the tumor cells [48]. Brefeldin A treatment, an inhibitor of COPI vesicles, inhibits the secretion of vascular endothelial growth factor receptor 1 and damages blood vessel sprouting and morphogenesis [75]. Therefore, the role of impaired transport function caused by COPI vesicle inhibition in autophagy requires further consideration.

In summary, we have shown that COPI regulates osteogenic effects in an autophagy-dependent way. This study uncovers a new function of COPI vesicles in regulating autophagy and provides additional insights into the mechanisms of mineralization.

## 4. Materials and Methods

### 4.1. Animals, Osteoporosis Model and COPI Dysfunction by β-COP siRNA

For the osteoporosis model, female Sprague Dawley rats (*n* = 12, 9–10 weeks old) were purchased from the Animal Center of Xi’an Jiaotong University (Xi’an, China). The rats were randomly separated into the control group (*n* = 3) and osteoporosis group (*n* = 3). The osteoporosis group rats were administered retinoic acid (A9120, Solarbio, Beijing, China) at 75 mg/kg body weight each day for two weeks via intragastric administration as previously described [35]. The control group rats were administered normal saline (IN9000, Solarbio, Beijing, China), also known as 0.9% NaCl. COPI dysfunction in the rats was established by β-COP siRNA in the osteoporosis model, and the siRNA transfection complex was prepared according to the manufacturer’s instructions. The specific β-COP siRNA (sense-GGAGAUGUAAAGUCAAAGA, antisense-UCUUUGACUUUACAUCUCC) and control scramble siRNA were synthesized (Tsingke, Beijing, China). Briefly, 5 nmol of siRNA, 40 μL of in vivo transfection reagent (IVF3001, Invitrogen, Waltham, MA, USA), and 40 μL of 10% glucose were mixed for 15 min at room temperature. The β-COP siRNA (*n* = 3) or scrambled siRNA (*n* = 3) complexes were injected through medullary cavity injection of proximal tibias twice over the course of 4 weeks as described [76,77]. All experiments were carried out in accordance with the Guide for the Care and Use of Laboratory Animals. 

### 4.2. micro-CT Imaging and Analysis

Tibias dissected from rats were fixed in 4% paraformaldehyde for 48 h. The samples were analyzed by high-resolution micro-CT (PerkinElmer, Waltham, MA, USA). The scanner was set to 90 kV and 88 μA at 18 μm resolution. The region of interest (ROI) was defined as the area between 2 and 7% proximal to the growth plate in the distal tibia as described [21]. QuantumGX (PerkinElmer) was used to perform the reconstruction process, and Analyze (PerkinElmer) was used to perform the quantitative analysis in accordance with the guidance of the American Society for Bone and Mineral Research [78].

### 4.3. Histomorphological Analysis and Immunohistochemistry Staining (IHC)

The bone samples were obtained from the right femur of the rats and fixed in 4% paraformaldehyde (P1110, Solarbio, Beijing, China) for 48 h at room temperature (RT). Subsequently, those samples were decalcified in 10% EDTA solution (E1171, Solarbio, Beijing, China) over 4 weeks in a cradle at RT. Tissues were dehydrated, embedded in paraffin, and cut into 8.0 μm sections. The sections were treated with Goldner staining (G3550, Solarbio, Beijing, China) and H&E (G1120, Solarbio, Beijing, China). For IHC, the slices were incubated with the first antibody overnight and then nurtured with a secondary antibody for 1 h at RT the next day. The samples were incubated with streptavidin–biotin complex SABC-AP (SA1020, Boster, Wuhan, China). The primary antibodies LC3B (A19665, ABclonal, Wuhan, China), BECLIN1 (A21695, ABclonal, Wuhan, China), α-COP (A19651, ABclonal, Wuhan, China), and β-COP (ab2899, Abcam, Boston, MA, USA) were used. All experiments were carried out following the manufacturer’s standard protocol. The images were obtained and analyzed by “image J v1.8.0” software.

### 4.4. Cell Culture, Osteogenic Induction and Transfection

MG63 (CL-0157, Procell, Wuhan, China) cells were purchased. Cells were cultured in Dulbecco’s Modified Eagle Medium (DMEM) (11965092, Gibco, Invitrogen, Waltham, MA, USA), supplemented with 10% fetal bovine serum (FBS) (10091148, Gibco, Invitrogen, Waltham, MA, USA) and 1% penicillin-streptomycin (C0222, Beyotime, Shanghai, China). Cells were maintained at 37 °C, 5% CO_2_ atmosphere. For osteogenic induction, cells were cultured with osteogenic differentiation medium (ODM), comprising 10% FBS, 1% penicillin-streptomycin, 50 μM ascorbic acid (AX1775, Sigma-Aldrich, St. Louis, MO, USA), 10 mM β-glycerophosphate (G9422, Sigma-Aldrich, St. Louis, MO, USA), and 100 nM dexamethasone (D4902, Sigma-Aldrich, St. Louis, MO, USA). The culture medium was refreshed every 2–3 days. For knockdown of COPI, specific β-COP siRNA (sense-GGUCUGUCAUGCUAAUCCA, antisense-UGGAUUAGCAUGACAGACC) and control scramble siRNA were synthesized (Tsingke, Beijing, China). Transfections were performed using Lipofectamine 3000 Transfection Reagent (L3000075, Invitrogen, Waltham, MA, USA) according to the manufacturer’s protocol.

### 4.5. Western Blot Analysis

The cells were lysed with RIPA lysis buffer (P0013B, Beyotime, Shanghai, China) and added to a protease inhibitor cocktail (P1005, Beyotime, Shanghai, China). The total protein extracts were obtained after centrifugation at 12,000 rpm for 5 min at 4 °C and quantitated using a BCA protein assay kit (P0009, Beyotime, Shanghai, China). Then, cell extracts were denatured after the addition of 5 × SDS sample loading buffer (P0015, Beyotime, Shanghai, China) and boiled at 100 °C for 10 min. The total proteins are separated by SDS–PAGE. The target proteins were transferred to PVDF membranes (IPVH00005, Merck Millipore, Middlesex County, MA, USA), blocked with 5% (*w*/*v*) skim milk, and incubated with primary antibodies at 4 °C for 16 h. Those PVDF membranes were incubated with matched secondary antibodies at RT for 1 h. An ECL-enhanced chemiluminescence substrate kit (C0712, Merck Millipore, Middlesex County, MA, USA) was used to visualize immunoreactive bands. The primary antibodies α-COP (A19651, ABclonal, Wuhan, China), β-COP (ab2899, Abcam, Boston, MA, USA), LC3B (14600-1-AP, Proteintech, Wuhan, China), ALP (sc-271431, Santa Cruz, Dallas, TX, USA), RUNX2 (D1L7F, Cell Signaling Technology, Danvers, MA, USA), S6K1 (A4898, ABclonal, Wuhan, China), p-S6K1 (AP1389, ABclonal, Wuhan, China), BECLIN1 (A21695, ABclonal, Wuhan, China) and GAPDH (60004-1-Ig, Proteintech, Wuhan, China) were used. Protein expression was quantified using ImageJ v1.8.0 software.

### 4.6. Immunofluorescence (IF) Analysis and GFP-LC3 Assay

Cells were grown on 14 mm coverslips at a density of 1 × 10^4^ cells/well in 24-well plates. Samples were washed with fresh PBS and fixed with 4% paraformaldehyde for 20 min at RT, followed by a rinse with PBS. Then, cells performed permeabilization of 0.1% Triton X-100 (P0096, Beyotime, Shanghai, China) for 15 min and were blocked with 5% BSA in PBS plus 0.1% Tween-20 (ST671, Beyotime, Shanghai, China) for 30 min at RT. The primary antibody, diluted with 5% BSA in PBS plus 0.1% Tween-20, was used for incubation with samples at 4 °C for 16–18 h. The coverslips were washed 3 times and nurtured with diluted Alex Fluor conjugated secondary antibody in 5% BSA in PBS plus 0.1% Tween-20 at RT for 1 h. After washing with PBS plus 0.1% Tween-20 3 times, the nucleus was stained with DAPI (AR1176, Boster, Wuhan, China) at RT for 10 min. The primary antibodies LC3B (14600-1-AP, Proteintech, Wuhan, China), β-COP (sc-393615, Santa Cruz, Dallas, TX, USA), WIPI2 (28820-1-AP, Proteintech, Wuhan, China), and FIP200 (17250-1-AP, Proteintech, Wuhan, China) were used. The corresponding fluorescent secondary antibodies were used. For the GFP-LC3 assay, cells were transfected with GFP-LC3 plasmids (AD202001, Vigene Biosciences, Jinan, China) at a multiplicity of infection (MOI) = 200. Cells were fixed and labeled with DAPI. Images were acquired by a confocal microscope (FV3000, Olympus, Tokyo, Japan). Image analysis was performed by the software “Fiji v2.9.0”. To analyze the co-localization index, the plugin “Coloc 2” was used to obtain Spearman’s coefficient.

### 4.7. Alkaline Phosphatase (ALP) and Alizarin Red S Staining

After the stimulation of ODM, cells were rinsed with PBS 3 times and fixed with 4% paraformaldehyde for 20 min at RT. Then, cells were stained with alkaline phosphatase (ALP) staining solution (P0321S, Beyotime, Shanghai, China) at RT for 30 min. Images were taken and densitometry analysis was performed using ImageJ v1.8.0 software. The other cells were similarly treated and stained with Alizarin Red S staining solution (A5533, Sigma-Aldrich, St. Louis, MO, USA) for 10 min in a dark environment. For quantitative analysis, samples were destained with 10% (*v*/*v*) cetylpyridinium chloride monohydrate (C0732, Sigma-Aldrich, St. Louis, MO, USA) for 30 min. Absorbance was measured at 560 nm.

### 4.8. Cell Counting Kit-8 (CCK-8) Assay

Cells were grown at a density of 0.5 × 10^4^ cells/well in 96-well plates. The Cell Counting Kit-8 (AR1199, Boster, Wuhan, China) assay was conducted according to the manufacturer’s instructions. Absorbance was measured at 450 nm.

### 4.9. mRNA Extraction and qPCR

Total RNA extraction was performed on ice using TRIzol Reagent (Invitrogen, Waltham, MA, USA), according to the manufacturer’s protocols. After the extraction, reverse transcription of the RNA was carried out for cDNA synthesis using a Reverse Transcription Reagent (Invitrogen, Waltham, MA, USA). qPCR was performed using an SYBR Green PCR reagent kit (Roche, Basel, Switzerland) by a Real-Time PCR System (Applied Biosystems, Carlsbad, CA, USA). Relative mRNA expression of the target genes was normalized against that of GAPDH using the 2−ΔΔCt method. The oligonucleotide primers are shown: *GAPDH* (Forward Primer: GCCTGGAGAAACCTGCCAA, Reverse Primer: CCCTGTTGCTGTAGCCGTAT) and *β-COP* (Forward Primer: GAAAGAGCTCGTTTTATTCGCT, Reverse Primer: CCAGATCCTGTAGTACTCGTTC).

### 4.10. Statistical Analysis

Densitometry analysis was accomplished using ImageJ v1.8.0 software. Statistical analyses and visual representation were achieved using GraphPad Prism based on normally distributed datasets with equal variance (Bartlett’s test). Statistical significances were defined using an unpaired Student’s *t*-test and one-way analysis of variance (ANOVA) based on Tukey’s multiple comparisons post hoc test unless stated otherwise. Each point value represents mean ± SD values unless otherwise stated. Significance is defined as * *p* < 0.05, ** *p* < 0.01, *** *p* < 0.001, and ns—not significant. 

## 5. Conclusions

In this study, we found that COPI vesicles regulated autophagy via the mTORC1 pathway in mineralization. The levels of COPI vesicles were elevated in osteogenesis, and the disruption of COPI vesicles led to impaired osteogenic effects on account of the suppression of autophagic activity. The inhibition of mTORC1 rescues the suppression of COPI vesicle disruption of osteogenic capacity. Our study provides a new perspective on the underlying mechanism of COPI vesicle–autophagy interplay in mineralization.

## Data Availability

The datasets used or analyzed during the current study are available from the corresponding author upon reasonable request.

## Supplementary Materials

The following supporting information can be downloaded at: https://www.mdpi.com/article/10.3390/ijms25010339/s1.
