# Supplementary material for: COPI Vesicle Disruption Inhibits Mineralization via mTORC1-Mediated Autophagy
Source: Int J Mol Sci. 2023 Dec 26;25(1):339. doi: 10.3390/ijms25010339 (PMC10779376; doi:10.3390/ijms25010339)
Supplement: Supplementary file 1 [file ijms-25-00339-s001.zip › Supplementary Materials.pdf]

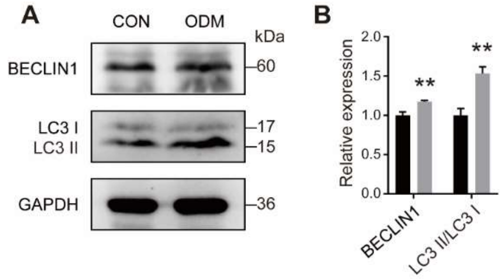

Figure S1. Autophagy activity is elevated after osteogenic induction. (A and B) Western blot and quantitative analysis of autophagic proteins (BECLIN1 and LC3). n = 3 per group. Data are means ± SD, and *P* values were quantified using t test. $^*P < 0.05$; $^{**}P < 0.01$.

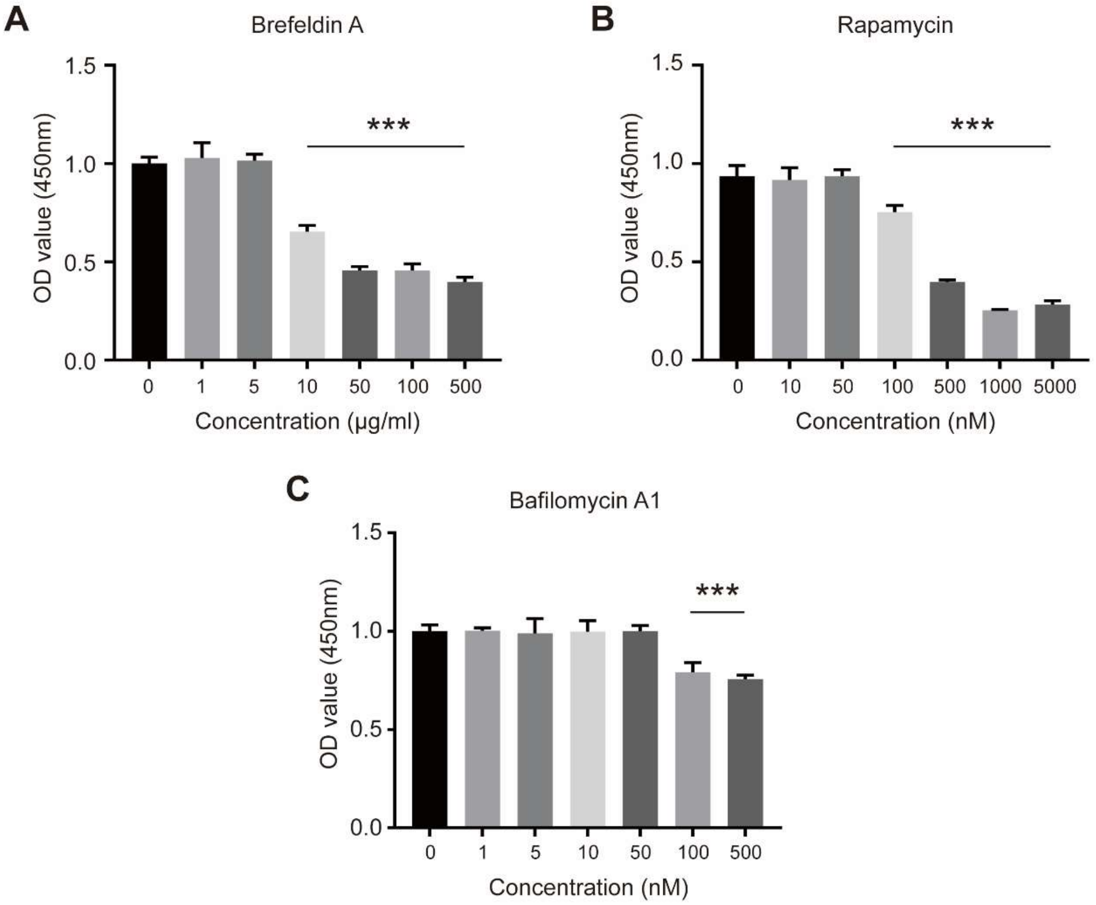

Figure S2. Cytotoxicity evaluations of the viability when MG63 exposed to gradient Brefeldin A, Rapamycin and Bafilomycin A1. (A and B) CCK-8 assay of MG63 after exposure to gradient Brefeldin A (A), Rapamycin (B) and Bafilomycin A1 (C). n = 5 per group. Data were presented as mean ± SEM, and *P* values were quantified using one-way ANOVA with Tukey's post hoc test. $^{***}P < 0.001$.

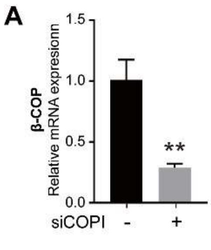

Figure S3. The expression of β-COP is decreased in siCOPI cells. (A) qPCR of β-COP in siCOPI cells. n

=3 per group. Data are means ± SD, and *P* values were quantified using t test. ***P* < 0.01.

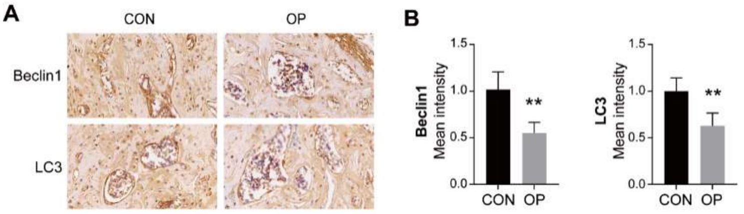

Figure S4. Autophagy activity is descended in osteoporosis. (A and B) Representative immunohistochemical images of distal femora sections staining with autophagic proteins (Beclin1 and LC3) and quantitative analysis of those proteins (B) from CON (control) and OP (osteoporosis) rats. n = 3 per group. Data are means ± SD, and *P* values were quantified using t test. ***P* < 0.01.
