# Supplementary figures and images for: COPI Vesicle Disruption Inhibits Mineralization via mTORC1-Mediated Autophagy
Source: Int J Mol Sci. 2023 Dec 26;25(1):339. doi: 10.3390/ijms25010339 (PMC10779376; doi:10.3390/ijms25010339)

Figure 2C

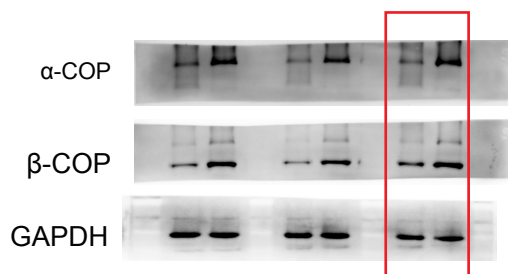

Figure 2G and 2K

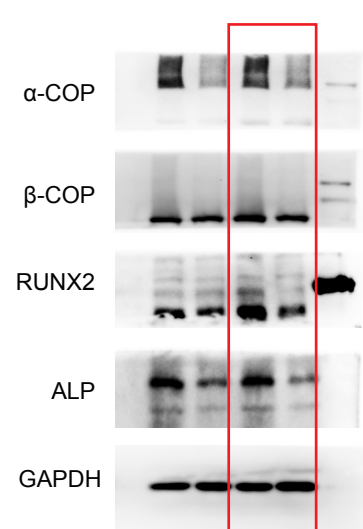

FigureS 1A

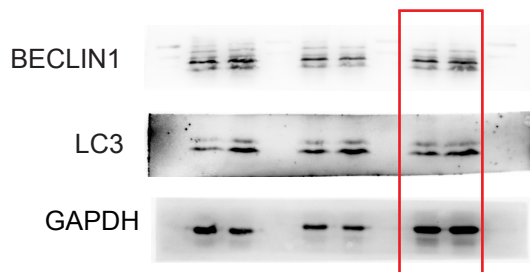

Figure 3L

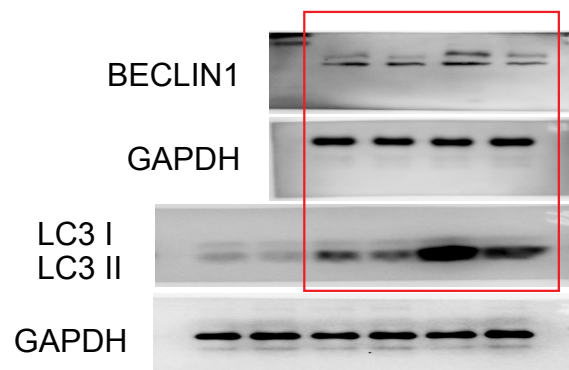

Figure 3J

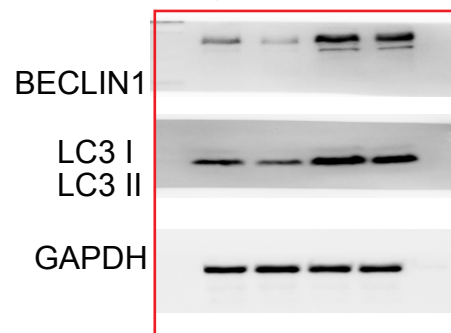

Figure 4C

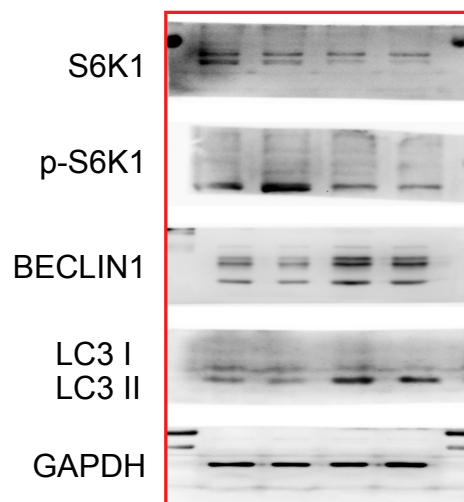

Figure 4E

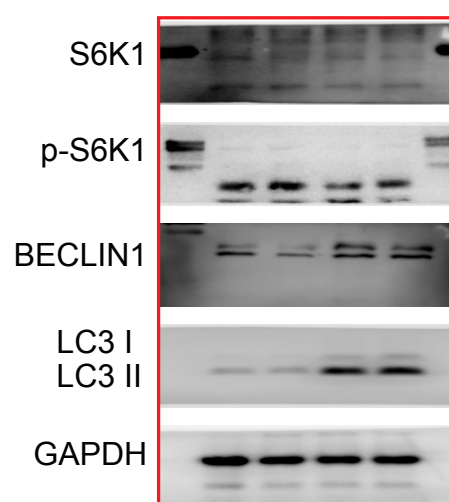

Figure 5A

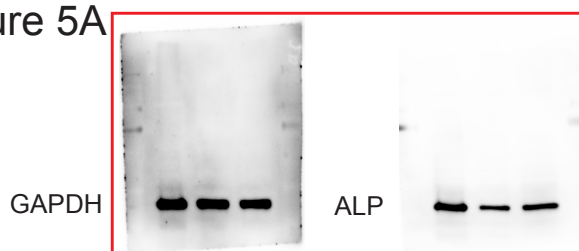

Figure 5A

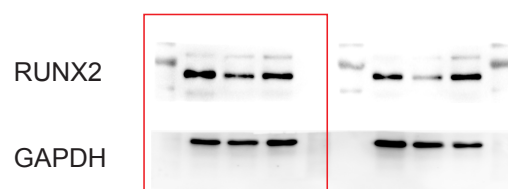

Supplement: Supplementary file 1 [file ijms-25-00339-s001.zip › Western blot results.pdf]
